# Impact of discontinuation of contact precautions on surveillance- and whole genome sequencing-defined methicillin-resistant *Staphylococcus aureus* healthcare-associated infections

**DOI:** 10.1017/ash.2024.89

**Published:** 2024-06-04

**Authors:** Sharon Karunakaran, Lora Lee Pless, Ashley M. Ayres, Carl Ciccone, Joseph Penzelik, Alexander J. Sundermann, Elise M. Martin, Marissa P. Griffith, Kady Waggle, Jacob C. Hodges, Lee H. Harrison, Graham M. Snyder

**Affiliations:** 1 Division of Pediatric Infectious Diseases, Children’s Hospital of Pittsburgh, Pittsburgh, PA, USA; 2 Division of Infectious Diseases, University of Pittsburgh School of Medicine, Pittsburgh, PA, USA; 3 Microbial Genomics Epidemiology Laboratory, Center for Genomic Epidemiology, University of Pittsburgh, Pittsburgh, PA, USA; 4 Department of Infection Prevention and Control, UPMC Presbyterian/Shadyside, Pittsburgh, PA, USA; 5 Veterans’ Affairs Pittsburgh Healthcare System, Pittsburgh, PA, USA; 6 The Wolff Center at UPMC, Pittsburgh, PA, USA

## Abstract

**Objective:**

Prior studies evaluating the impact of discontinuation of contact precautions (DcCP) on methicillin-resistant *Staphylococcus aureus* (MRSA) outcomes have characterized all healthcare-associated infections (HAIs) rather than those likely preventable by contact precautions. We aimed to analyze the impact of DcCP on the rate of MRSA HAI including transmission events identified through whole genome sequencing (WGS) surveillance.

**Design:**

Quasi experimental interrupted time series.

**Setting:**

Acute care medical center.

**Participants:**

Inpatients.

**Methods:**

The effect of DcCP (use of gowns and gloves) for encounters among patients with MRSA carriage was evaluated using time series analysis of MRSA HAI rates from January 2019 through December 2022, compared to WGS-defined attributable transmission events before and after DcCP in December 2020.

**Results:**

The MRSA HAI rate was 4.22/10,000 patient days before and 2.98/10,000 patient days after DcCP (incidence rate ratio [IRR] 0.71 [95% confidence interval 0.56–0.89]) with a significant immediate decrease (*P* = .001). There were 7 WGS-defined attributable transmission events before and 11 events after DcCP (incident rate ratio 0.90 [95% confidence interval 0.30–2.55]).

**Conclusions:**

DcCP did not result in an increase in MRSA HAI or, in WGS-defined attributable transmission events. Comprehensive analyses of the effect of transmission prevention measures should include outcomes specifically measuring transmission-associated HAI.

## Background

Current guidelines recommend using contact precautions (CP) to prevent methicillin-resistant *Staphylococcus aureus* (MRSA) transmission and infection in acute care hospitals.^1,2^ Following published reports of discontinuation of contact precautions (DcCP), a decreasing proportion of hospitals report using CP for MRSA. Although high-quality studies of universal CP or a bundle of MRSA control interventions suggest an inverse relationship between CP and infections due to MRSA,^3,4^ many observational and quasi-experimental studies characterizing the impact of DcCP have universally shown no impact on MRSA healthcare-associated infections (HAIs) after DcCP.^5–7^

However, a common limitation of these studies is the non-specificity of the HAI outcome: The facility-wide rate of MRSA HAI does not distinguish between HAI resulting from MRSA transmission in the hospital and HAI occurring due to a non-hospital acquired endogenous pathogen.^8,9^ CP are most likely to have a demonstrable preventative effect on MRSA HAI resulting from transmission during hospitalization, in contrast to HAI resulting from the patients’ own endogenous flora. Focusing on the outcome of probable transmission events may improve the accuracy of the estimated impact of CP in preventing transmission. Whole genome sequencing (WGS) allows more accurate characterization of healthcare-associated transmission events, including events that may not be characterized as HAI.^10,11^ Utilizing WGS-supported data may confirm or refute the conclusion that DcCP does not change the incidence of transmission-associated HAI events.

In this quasi experimental time series study, we characterized transmission events identified using WGS in the context of measuring the relationship between DcCP and the facility-wide MRSA HAI rate.

## Methods

### Setting

This quasi experimental time series study took place at UPMC Presbyterian, a 695-bed adult tertiary academic acute care hospital which provides Level 1 trauma, critical care, and organ transplant services, comprising 34 inpatient units; all inpatient units were included. The proportion of beds in a cohorted room (two beds per room) in the hospital is approximately 26% of inpatient beds.

All patients admitted to the hospital not known to have MRSA carriage are ordered to have admission and weekly active surveillance nares screening performed by culture for MRSA; median (interquartile range) facility-wide monthly adherence rates for admission and weekly swabs during the study periods were 80.6% (77.6, 84.2) and 55.9% (49.4, 60.6), respectively (Supplemental Figure 1), and the admission prevalence during the study period was 2.0%. The median (interquartile range) intensive care unit (ICU)-specific monthly adherence rates for admission and weekly swabs during the study periods were 91.5% (87.6, 94.9) and 30.6% (20.2, 45.6), respectively (Supplemental Figure 1), and the admission prevalence during the study period was 2.3%. A patient’s MRSA carriage indicator in the electronic health record could be removed if the following criteria were met: (1) no less than 30 days had passed since the last positive culture (surveillance or clinical culture), (2) at least 7 days had passed since receipt of antimicrobial with activity against MRSA, and (3) three consecutive negative nares cultures were collected at least 24 hours apart. Prior to the intervention, patients with current or prior MRSA carriage (colonized or infected) were admitted to a single occupancy room or were cohorted with another patient with concurrent or prior MRSA carriage; subsequent to the intervention, MRSA carriage did not affect bed placement. Other infection prevention approaches potentially affecting MRSA outcome ascertainment and transmission did not change in the study period (Supplemental Table 1).

### Study design

This study measures MRSA HAI outcomes before and after DcCP in December 2020, including a pre-intervention period January 2019 through November 2020 and a post-intervention period December 2020 through December 2022. Contact precautions—using gown and gloves for all care involving patient contact and in patient’s environment—were used for patients identified as MRSA carriers until December 2020 after which they were no longer required for care of patients with prior or current MRSA infections or colonization. No units were excluded from DcCP. Before and after the intervention, as part of standard precautions, use of gowns and gloves were recommended for any potential contact with blood or body fluids including draining wounds. The use of CP for vancomycin-resistant enterococci (VRE) among carriers, and VRE active surveillance for all patients, was employed during the entire study period. During the post-intervention period, VRE and MRSA co-infection was noted in two patients, hence requiring use of CP.

We measured two co-primary outcomes: National Healthcare Safety Network (NHSN) reported MRSA HAI rates and the frequency of WGS-confirmed MRSA transmission events. NSHN HAI were defined for all HAI types using NHSN procedures.^12,13^ WGS surveillance was performed on MRSA isolates from clinical specimens identified ≥3 days after hospitalization or within 30 days of a prior healthcare exposure. Isolates from asymptomatic screening were not included. DNA was extracted from patient isolates using MagMAX DNA extraction kit on King Fisher Apex and sequencing libraries were prepared using Illumina DNA Prep Tagmentation kit on an Eppendorf EpMotion liquid handler per manufacturer’s instructions. Samples were pooled in equimolar concentration and then sequenced on an Illumina NextSeq 550 using the v2.5, 300 cycle kit. The resulting WGS reads were assembled using Unicycler v0.5.0,^14^ annotated using Prokka v1.14,^15^ and species were determined using Kraken2.^16^ Transmission clusters were determined by computing the pairwise single nucleotide polymorphisms (SNPs) among the patient isolates of the same species in the database (restricted to the period WGS was performed) using Snippy v4.3.0^17^ or SKA v1.0.^18^ Isolate pairs with 15 or fewer pairwise core genome SNP differences were considered genetically related, representing transmission.^19^ WGS events were considered *attributable* to the study period if the isolate was the second or subsequent genetically related isolate with a “source” isolate during the study period (including pre-DcCP or post-DcCP periods). The related isolates were also examined for geo-temporal and procedural commonalities: transmission events for which the patient pair were cared for on the same unit with at least one day of overlap on the unit, or who received care from a specific healthcare worker concomitantly, were considered epidemiologically linked.

**Figure 1.**
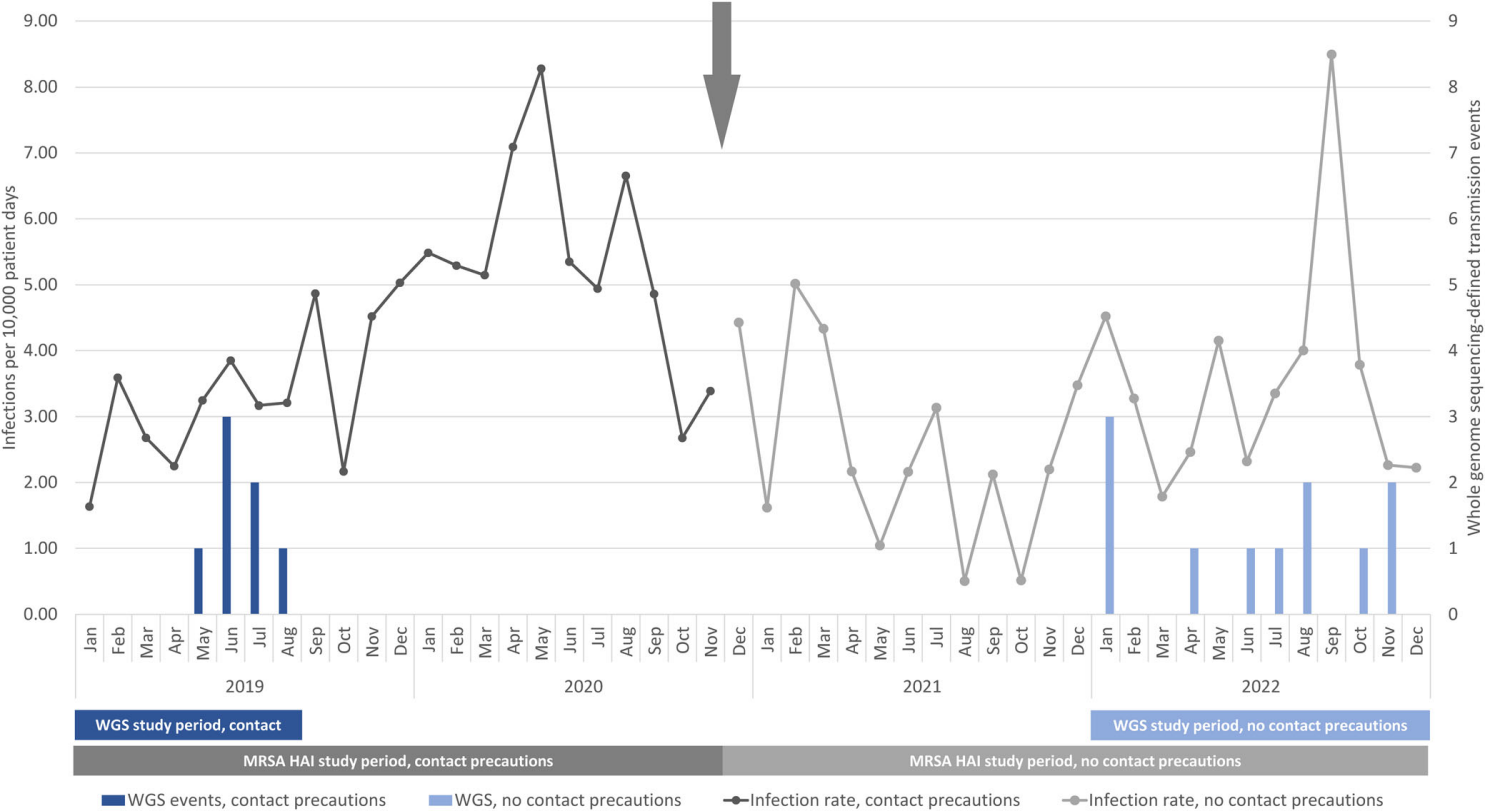
The frequency of co-primary outcomes of methicillin-resistant *Staphylococcus aureus* healthcare-associated infections and whole genome sequencing-defined transmission events before and after discontinuation of contact precautions. Note: WGS, whole genome sequencing. Arrow indicates date of study intervention.

WGS was performed as part of a preexisting research investigation in two different stages: Isolates were banked and analyzed retrospectively until August 2019, and from January 2022 onward WGS was performed in “real time” with infection preventionist notification of genetically related pairs.^19,20^ At the time of the current analysis, isolates from September 2019 to December 2021 were not sequenced. Therefore, the analysis periods for WGS-confirmed transmission were restricted to the availability of WGS data during the study periods: January through August 2019 during the use of CP and January through December 2022 after DcCP (Figure 1).

This investigation was approved (Project 4222) as a quality improvement project by the UPMC Quality Improvement Review Committee.

### Statistical analysis

To evaluate the impact of DcCP on HAI rates, we performed a time series analysis of MRSA HAI rates before and after DcCP, using a linear regression model to calculate the step change and change in slope. The frequency of subcategories of HAI in each study period were descriptive in nature. To compare the change in HAI and WGS-defined attributable transmission events, we calculated an incidence rate ratio (IRR), 95% confidence intervals (95%CI), and *P* values using a median-unbiased estimation. Characteristics of WGS-defined transmission events were descriptive. Each co-primary outcome was calculated per 10,000 patient days; as a sensitivity analysis, we calculated these outcomes per 1,000 patient admissions.

We also performed several exploratory analyses. Because MRSA transmission and HAI is more common in ICUs than non-ICUs,^21^ we analyzed HAI attributed to ICU locations per 10,000 critical care patient days. One estimate of transmission is acquisition of asymptomatic carriage in individuals without prior MRSA carriage; therefore, we calculated the new acquisition rate per 10,000 patient days as measured using by routine active surveillance. Since CP did not change for VRE during the study period, we calculated the rate of VRE HAI per 10,000 patient days to serve as a non-intervention comparison. These rates were evaluated as time series analyses as described for the primary HAI outcome and qualitatively compared to the findings for the primary HAI outcome. All analyses were performed using Stata version 16.0 software (StataCorp, College Station, TX).

## Results

### Primary outcomes

There were 303 MRSA HAI during the study period, including 170 in the 23-month pre-intervention period during which CP were routinely used, and 133 in the 25-month post-intervention period after which CP were discontinued. The most common HAI types were surgical site infection (96, 31.7%), pneumonia (58, 19.1%), and skin/soft tissue infection (49, 16.2%) (Supplemental Table 2). During the entire study period, there were 129,588 admissions accounting for 849,741 patient days (Supplemental Table 3).

The MRSA HAI rates in the pre-intervention period were 4.22 HAI/10,000 patient days and 2.54 HAI/1,000 admissions, and in the post-intervention period the MRSA HAI rates were 2.98 HAI/10,000 patient days (IRR 0.71, 95%CI 0.56–0.89, *P* = .003) and 2.12 HAI/1,000 admissions (IRR 0.83, 95%CI 0.66–1.05, *P* = .12) (Figure 1). For the primary outcome of MRSA HAI per 10,000 patient days, there was a non-significant change in the trend in HAI rate before and after DcCP (*P* = .18) and a significant immediate decrease in the MRSA HAI rate (*P* = .001) at the time of DcCP.

Sequencing data were available for 111 clinical isolates in the pre-DcCP period and 276 isolates after DcCP (Supplemental Table 4). In the 8-month pre-intervention period, there were 11 cases related to ≥1 clinical isolate by WGS, of which 7 were attributable to transmission during the study period; 6 of these 7 cases were epidemiologically linked to another case during the pre-intervention period, defined as either concomitantly cared for on the same unit or by a specific healthcare provider during an overlapping period of time (Table 1). In the 12-month post-intervention period, there were 23 cases related to ≥1 clinical isolates by WGS, of which 11 were attributable to transmission during the study period; 5 of these 11 non-source cases were epidemiologically linked to another case during the post-intervention period. The IRR for the change in transmission events was 0.90 (95%CI, 0.30–2.55; *P* = .85) for the rate per 10,000 patient days and 0.72 (95%CI, 0.24–2.03; *P* = .51) for the rate per 1,000 admissions.

**Table 1.** Whole genome sequencing-defined methicillin-resistant *Staphylococcus aureus* transmission events occurring before and after discontinuation of contact precautions

Genetically related cluster	Culture date	Culture source	Attributable to transmission during the study period	Epidemiologically linked	Rationale
1	5/1/2019	Sputum	No	No	Source
1	6/7/2019	Wound	Yes	Yes	Geotemporal
1	6/12/2019	Sputum	Yes	Yes	Geotemporal
1	7/11/2019	Blood	Yes	Yes	Geotemporal
1	7/31/2019	Blood	Yes	Yes	Geotemporal
1	8/2/2019	Blood	Yes	Yes	Geotemporal
6	1/31/2019	Sputum	No	No	Source
6	5/17/2019	Wound	Yes	No	No overlap
7	4/19/2019	Wound	No	No	Source
7	6/2/2019	Blood	Yes	Yes	Geotemporal
8	7/8/2019	Unknown	No	No	Transmission in different study periods
2	1/7/2022	Tissue	No	No	Source
2	1/22/2022	Sputum	Yes	Yes	Geotemporal and healthcare worker
2	1/30/2022	Blood	Yes	Yes	Geotemporal and healthcare worker
3	3/20/2022	Sputum	No	No	Source
3	4/2/2022	Sputum	Yes	Yes	Geotemporal
3	4/27/2022	Sputum	No	No	Duplicate isolate
4	5/18/2022	Blood	No	No	Source
4	7/24/2022	Sputum	Yes	No	No overlap
4	8/11/2022	Blood	Yes	No	No overlap
5	9/15/2022	Blood	No	No	Source
5	10/16/2022	Wound	Yes	Yes	Geotemporal
5	11/7/2022	Wound	No	No	Duplicate isolate
8	11/14/2022	Wound	No	No	Transmission in different study periods
9	1/24/2022	Sputum	No	No	Source
9	1/31/2022	Sputum	Yes	Yes	Geotemporal
10	5/4/2022	Blood	No	No	Source
10	8/23/2022	Wound	Yes	No	No overlap
11	6/5/2022	Blood	No	No	Source
11	6/22/2022	Sputum	Yes	No	No overlap
12	9/29/2022	Blood	No	No	Source
12	11/11/2022	Unknown	Yes	No	Unknown
13	11/3/2022	Blood	No	No	Source
13	11/4/2022	Sputum	Yes	No	No overlap

*Note:* ICU, intensive care unit. “Geotemporal” refers to care for the source patients on the same unit during overlapping periods; “healthcare worker” indicates a common healthcare worker caring for both patients concomitantly (not necessarily on the same unit); “source” indicates the earliest genetically related isolate in the cluster (and therefore the first patient in a transmission event); “No overlap” indicates to identifiable epidemiological association; Patient care unit denotes the unit type at the time of clinical culture collection.

### Exploratory outcomes

There were 40 MRSA HAI attributed to ICU locations of care occurring during 80,414 ICU patient days in the pre-intervention period (4.97 HAI/10,000 patient days), and 25 MRSA HAI during 90,828 ICU patient days of care in the post-intervention period (2.75 HAI/10,000 patient days; IRR 0.55, 95%CI 0.32–0.93, *P* = .02) (Supplemental Table 3). The change in the trend in ICU MRSA HAI rate per 10,000 patient days before and after DcCP was non-significant (*P* = .79), and there was a significant immediate decrease in the ICU MRSA HAI rate (*P* = .04) at the time of DcCP (Supplemental Table 2, Supplemental Figure 2).

MRSA acquisition defined by new asymptomatic carriage on active surveillance nares cultures occurred 440 times during 402,999 patient days in the pre-intervention period and 542 times during 446,746 patient days in the post-intervention period (IRR 1.11, 95%CI 0.98–1.26, *P* = .10) (Supplemental Table 3). There was not a significant change in either the trend in asymptomatic MRSA acquisition rate before and after DcCP (*P* = .35), nor a significant immediate change in the asymptomatic MRSA acquisition rate (*P* = .62) at the time of DcCP (Supplemental Figure 3).

There were 155 VRE-attributed HAI in the pre-intervention period and 163 VRE-attributed HAI in the post-intervention period (IRR 0.95, 95%CI 0.76–1.19, *P* = .64) (Supplemental Table 3). There was a non-significant change in the trend in asymptomatic VRE acquisition rate before and after DcCP (*P* = .19) and a non-significant immediate change in the asymptomatic VRE acquisition rate (*P* = .55) at the time of DcCP (Supplemental Figure 4).

## Discussion

In this 48-month retrospective observation study, we characterized the impact of DcCP on the HAI and transmission rate of MRSA. We found a significant decrease in the incidence MRSA HAI from 4.22 to 2.98 HAI per 10,000 patient days, associated with a significant immediate decrease in the HAI rate but no significant difference in the HAI rate trend after implementation. Following DcCP, we did not observe a significant change in WGS-defined attributable transmission events, nor a significant change in acquisition defined by new asymptomatic carriage on active surveillance. These findings describe a more focused estimate of transmission and provide a model for more nuanced and causal assessment of the impact of transmission prevention measures (such as CP) as a subset of infection prevention measures.

Our study findings are consistent with other studies that found no significant increase in MRSA HAI rates after DcCP.^5^ We hypothesize, however, that not observing an increase in HAI may be because not all HAI result from pathogen acquisition while receiving care in the healthcare setting, and CP is an intervention focused on preventing transmission rather than preventing infection due to endogenous organisms.^22^ Therefore, a more causal assessment of the impact of CP on preventing HAI should focus on transmission, which may or may not result in clinically diagnosed infection. We propose that studies of interventions should include more direct measures of transmission, including using genetic relatedness of pathogens to identify transmission and new acquisition of carriage.^23,24^ Such outcomes will be particularly important to understand the benefit of CP not as an intervention either universally implemented or eschewed, but an intervention deployed with a risk-tailored approach.^25^

Our study is unique in assessing the causal relationship between DcCP and MRSA in an acute care hospital with a more comprehensive set of measures including NHSN-adjudicated HAI (including ICU-restricted analysis), WGS-validated transmission, asymptomatic acquisition identified by weekly nares screening, and a control organism with similar mechanism of transmission (VRE in this study). The reasons for a decrease and lower MRSA HAI incidence rate after DcCP overall and in the ICU-restricted analysis are unclear. Although we do not have robust data on adherence to standard or CPs, it is possible that after DcCP healthcare workers were more conscientious of transmission risk; hand hygiene adherence did not demonstrate an appreciable trend during the study period, though a formal assessment could not be undertaken due to a change in the method for measuring hand hygiene adherence at the study institution in August 2020. It may also be that other HAI prevention efforts not specific to MRSA may have improved HAI rates but not resulted in a change in transmission or acquisition. Temporal trends may also be influenced by the COVID-19 pandemic, which affected the complexity and mix of medical conditions for which people were hospitalized as well as the nature of care; however, this relationship has not been universally observed and infection prevention guidelines at the study institution did not change during the pandemic.^26–28^

Conclusions from this comprehensive approach of assessing the relationship between DcCP and MRSA transmission and infection should be tempered by the limitations of a single-center study with the particular characteristics of this facility such as proportion of shared beds, transmission prevention measures, and nature of care and patient complexity-and limitations of a pre-/post-observational study design. Our WGS data set was limited by the periods in which routine WGS surveillance was performed. Defining a MRSA isolate as genetically related and therefore attributed as transmission requires a prior isolate^29^; while we did not establish a baseline pool of potential sources, and isolates attributed to community acquisition were not sequenced, the limitation of misattribution should not differentially apply to the two study periods. As WGS becomes more accessible, future studies should broaden the transmission source population by including a baseline period of WGS before analysis of transmission, and include isolates presumed to have community acquisition that may serve as a source for transmission (and therefore increase ascertainment of transmission events). WGS in 2019 was retrospective, while WGS surveillance in 2022 was “real time” permitting infection preventionists to intervention when transmission was identified, which may also lead to a difference in study outcomes (HAI and WGS-defined transmission) not attributed to DcCP. The adherence to active surveillance was not 100% and adherence to weekly testing was qualitatively lower in the DcCP period; insufficient detection of asymptomatic carriage may make use of CP incomplete during the pre-DcCP period and provider knowledge of MRSA carriage may affect diagnostic testing practices (and therefore, case ascertainment). Our analysis only considers outcomes based on clinical isolates and does not estimate the effect of CP on preventing asymptomatic MRSA acquisition.

We evaluated NHSN-defined MRSA HAI hospital-wide and in ICUs, WGS-defined transmission events, and new asymptomatic acquisition of MRSA carriage, and NHSN-defined VRE HAI, before and after DcCP for MRSA. This analysis did not identify a significant increase in measures correlating with HAI, transmission, or acquisition outcomes. Although a robust scientific literature consistently reports no change in MRSA HAI after DcCP, future studies should replicate this more comprehensive methodology including the use of WGS to characterize transmission events when evaluating CP and other vertical infection prevention interventions, particularly when assessing risk-tailored approaches to the use of CP.

## Supporting information

Karunakaran et al. supplementary material Karunakaran et al. supplementary material

## Supplementary material

For supplementary material accompanying this paper visit http://doi.org/10.1017/ash.2024.89. click here to view supplementary material
