# Supplementary material for: Impact of discontinuation of contact precautions on surveillance- and whole genome sequencing-defined methicillin-resistant Staphylococcus aureus healthcare-associated infections
Source: Antimicrob Steward Healthc Epidemiol. 2024 Jun 4;4(1):e97. doi: 10.1017/ash.2024.89 (PMC11149034; doi:10.1017/ash.2024.89)

SUPPLEMENTAL MATERIALS

Karunakaran S, et al. *Impact of discontinuation of contact precautions on surveillance- and whole genome sequencing-defined methicillin-resistant Staphylococcus aureus healthcare-associated infections*

Table of Contents

Page(s)	Content
2	**Supplemental Table 1**. Infection prevention measures potentially related to methicillin-resistant *Staphylococcus aureus* at the study facility during the study period
3	**Supplemental Figure 1.** Adherence to methicillin-resistant *Staphylococcus aureus* active surveillance testing, during the study period
4	**Supplemental Table 2.** Healthcare-associated infection events, by month and infection type
5	**Supplemental Table 3.** Study outcome events (primary and secondary) and event rate denominators, by month
6	**Supplemental Table 4.** Clinical isolates available for sequencing and completed sequencing in the pre- and post-intervention periods
7	**Supplemental Figure 2.** Methicillin-resistant *Staphylococcus aureus* healthcare-associated infections in intensive care units, before and after discontinuation of contact precautions
8	**Supplemental Figure 3.** Methicillin-resistant *Staphylococcus aureus* acquisition by active surveillance, before and after discontinuation of contact precautions
9	**Supplemental Figure 4.** Vancomycin-resistant *Enterococcus sp* healthcare-associated infections, during the study period

Supplemental Table 1. Infection prevention measures potentially related to methicillin-resistant *Staphylococcus aureus* at the study facility during the study period

Infection prevention measure	Patient population	Pre-intervention	Post-intervention
Contact precautions	All patients with current or historical carriage.	Yes	No
Removing indicator of MRSA carrier status	Patients may be no longer identified as MRSA carriers following a standardized protocol with serial active surveillance (initiated at provider discretion).	Yes	Yes
MRSA active surveillance, admission	All inpatient admissions without prior MRSA carriage	Yes	Yes
MRSA active surveillance, pre-operative	Scheduled neurosurgical and orthopedic procedures	Yes	Yes
MRSA decolonization	Pre-operative *Staphylococcus aureus* carriers, neurosurgical and orthopedic procedures	Yes	Yes
Chlorhexidine skin treatment	All inpatients (without a contraindication) daily.<br>All scheduled surgical procedures that use onsite pre-operative testing.	Yes	Yes

Supplemental Figure 1. Adherence to methicillin-resistant *Staphylococcus aureus* active surveillance testing, during the study period

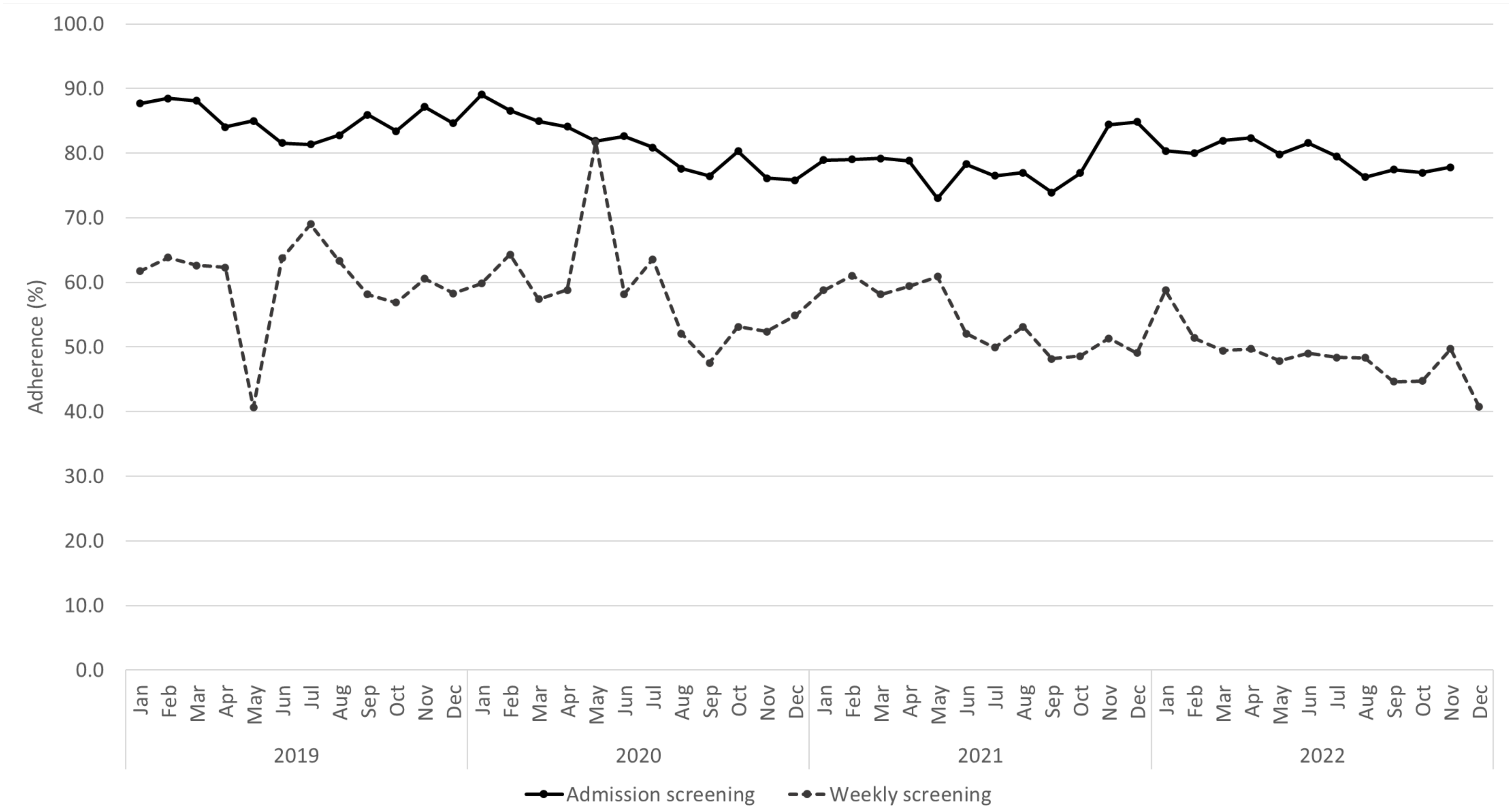

Supplemental Table 2. Healthcare-associated infection events, by month and infection type

		NHSN-defined HAI type												
Year	Month	BJ	BSI	CNS	CVS	EENT	GI	LRI	PNEU	SSI	SST	UTI	VAE	*Total*
	Jan	0	0	0	0	0	0	0	1	0	1	1	0	*3*
	Feb	2	1	0	1	0	1	0	0	1	0	0	0	*6*
	Mar	0	0	0	0	0	0	0	1	4	0	0	0	*5*
	Apr	0	0	0	0	0	0	0	0	1	2	0	1	*4*
	May	1	0	0	0	0	0	0	1	3	0	0	1	*6*
2019	Jun	0	0	0	0	0	0	0	2	3	2	0	0	*7*
	Jul	0	0	0	0	0	0	1	1	1	3	0	0	*6*
	Aug	0	0	1	0	0	0	0	2	3	0	0	0	*6*
	Sep	0	0	0	0	0	0	0	1	4	2	1	1	*9*
	Oct	0	0	0	0	0	0	0	2	1	1	0	0	*4*
	Nov	0	3	0	1	0	0	0	0	3	0	0	1	*8*
	Dec	0	4	0	0	0	0	0	2	1	0	2	0	*9*
	Jan	0	0	0	0	2	0	0	1	4	2	0	1	*10*
	Feb	0	1	0	0	0	0	0	2	3	1	1	1	*9*
	Mar	0	0	0	0	1	1	1	0	1	4	0	0	*8*
	Apr	0	3	0	0	0	0	1	0	2	2	0	0	*8*
	May	0	2	0	0	0	0	0	2	5	0	1	2	*12*
	Jun	0	0	1	0	0	0	0	2	4	2	0	0	*9*
	Jul	3	2	0	0	0	0	0	1	1	0	2	0	*9*
	Aug	1	3	0	0	0	0	0	3	4	0	0	1	*12*
	Sep	0	0	0	1	0	1	0	4	1	2	0	0	*9*
	Oct	0	0	0	0	0	0	0	1	2	1	0	1	*5*
	Nov	1	2	0	0	0	0	0	0	3	0	0	0	*6*
	Dec	0	0	1	0	0	1	0	2	3	1	0	0	*8*
	Jan	0	1	0	0	0	0	0	2	0	0	0	0	*3*
	Feb	0	2	0	1	0	0	0	2	1	2	0	0	*8*
	Mar	1	0	0	0	0	0	0	0	3	2	0	2	*8*
	Apr	0	0	0	0	0	0	0	2	2	0	0	0	*4*
	May	0	0	0	0	0	0	0	0	2	0	0	0	*2*
2021	Jun	0	2	0	0	0	0	0	0	2	0	0	0	*4*
	Jul	0	1	0	0	0	0	0	0	1	2	0	2	*6*
	Aug	0	0	0	1	0	0	0	0	0	0	0	0	*1*
	Sep	0	0	0	0	0	0	0	2	1	0	0	1	*4*
	Oct	0	0	0	0	0	0	0	0	0	1	0	0	*1*
	Nov	0	0	0	0	0	0	0	1	2	1	0	0	*4*
	Dec	1	0	0	0	0	0	1	0	1	3	0	0	*6*
	Jan	0	1	0	1	0	0	0	2	1	2	0	1	*8*
	Feb	0	1	0	0	0	0	0	0	3	1	0	0	*5*
	Mar	0	0	0	0	0	0	0	1	1	1	0	0	*3*
	Apr	0	1	0	0	0	0	0	1	1	1	0	0	*4*
	May	0	0	0	1	0	0	0	2	2	1	1	0	*7*
2022	Jun	0	0	0	2	0	0	0	1	1	0	0	0	*4*
	Jul	0	0	0	0	0	0	0	0	5	0	0	1	*6*
	Aug	0	0	0	0	0	0	0	2	1	2	1	1	*7*
	Sep	2	0	0	1	0	1	0	2	4	4	0	1	*15*
	Oct	0	0	0	0	0	0	0	6	1	0	0	0	*7*
	Nov	0	0	0	0	0	0	0	1	1	0	0	2	*4*
	Dec	0	1	0	0	0	0	0	0	2	0	0	1	*4*
Total		12	31	3	10	3	5	4	58	96	49	10	22	*303*
Pre-intervention		8	21	2	3	3	3	3	29	55	25	8	10	*170*
Post-intervention		4	10	1	7	0	2	1	29	41	24	2	12	*133*

Supplemental Table 3. Study outcome events (primary and secondary) and event rate denominators, by month

Year	Month	Admissions	**PatientDays**	Admissions_ICU	Admissions-NonICU	PatientDays_ICU	PatientDays_NonICU	**HAI**	HAI_ICU	**WGSevents**	*HAIrateAdmit_ICU*	HAI_VRE	Surveilance_tested	Surveillance_pos	Surveillance_acq
	Jan	3150	18358	564	2586	3650	14708	3	0	0	0.00	7	2466	57	13
	Feb	2908	16728	556	2352	3283	13445	6	2	0	3.60	6	2378	58	14
	Mar	3113	18655	573	2540	3913	14742	5	0	0	0.00	5	2588	55	24
	Apr	3037	17797	575	2462	3724	14073	4	1	0	1.74	7	2329	68	25
	May	3216	18514	591	2625	3655	14859	6	2	1	3.38	8	2492	71	21
2019	Jun	3086	18193	633	2453	3687	14506	7	2	3	3.16	4	2445	70	23
	Jul	3093	18957	581	2512	3777	15180	6	1	2	1.72	9	2389	60	23
	Aug	3236	18722	604	2632	3627	15095	6	2	1	3.31	9	2504	54	17
	Sep	3055	18489	629	2426	3708	14781	9	2		3.18	8	2361	46	16
	Oct	3236	18462	623	2613	3687	14775	4	2		3.21	8	2475	54	16
	Nov	2937	17701	552	2385	3532	14169	8	1		1.81	10	2389	50	15
	Dec	3053	17883	580	2473	3513	14370	9	2		3.45	5	2267	60	15
	Jan	3089	18223	556	2533	3552	14671	10	4		7.19	4	2401	100	38
	Feb	2842	17005	540	2302	3282	13723	9	2		3.70	3	2273	71	19
	Mar	2448	15541	478	1970	2881	12660	8	2		4.18	6	1925	71	17
	Apr	1859	11283	455	1404	2293	8990	8	1		2.20	5	1364	51	16
	May	2489	14501	485	2004	3076	11425	12	2		4.12	8	1800	53	17
2020	Jun	2825	16817	511	2314	3467	13350	9	0		0.00	5	2020	75	18
	Jul	2946	18211	554	2392	3515	14696	9	1		1.81	9	2140	67	20
	Aug	2845	18039	514	2331	3611	14428	12	6		11.67	4	2052	72	19
	Sep	2882	18516	588	2294	3646	14870	9	4		6.80	10	1815	62	13
	Oct	2899	18670	546	2353	3744	14926	5	1		1.83	9	2116	68	23
	Nov	2643	17734	490	2153	3591	14143	6	0		0.00	5	1834	65	18
	Dec	2581	18065	520	2061	3693	14372	8	2		3.85	9	1762	81	30
	Jan	2526	18542	475	2051	3552	14990	3	1		2.11	10	1784	48	13
	Feb	2387	15941	433	1954	3319	12622	8	2		4.62	6	1625	42	14
	Mar	2932	18464	540	2392	3417	15047	8	2		3.70	9	1956	57	16
	Apr	2845	18439	550	2295	3716	14723	4	0		0.00	13	1923	63	12
	May	2860	19141	573	2287	3812	15329	2	0		0.00	5	2012	49	17
2021	Jun	2858	18502	575	2283	3530	14972	4	1		1.74	4	1977	65	21
	Jul	2921	19154	579	2342	3698	15456	6	3		5.18	5	2089	85	27
	Aug	2738	19667	557	2181	3882	15785	1	0		0.00	5	1860	76	30
	Sep	2619	18831	565	2054	3725	15106	4	1		1.77	10	1766	81	26
	Oct	2559	19283	563	1996	3851	15432	1	0		0.00	4	1769	62	20
	Nov	2454	18176	532	1922	3529	14647	4	0		0.00	8	1590	74	31
	Dec	2403	17271	496	1907	3435	13836	6	0		0.00	7	1715	59	24
	Jan	2222	17688	483	1739	3598	14090	8	3	3	6.21	4	1473	41	20
	Feb	2086	15282	425	1661	3168	12114	5	3	0	7.06	5	1389	35	18
	Mar	2356	16800	472	1884	3433	13367	3	0	0	0.00	5	1435	51	7
	Apr	2393	16229	527	1866	3382	12847	4	1	1	1.90	9	1635	61	21
	May	2475	16856	518	1957	3496	13360	7	3	0	5.79	7	1553	49	22
2022	Jun	2339	17230	528	1811	3605	13625	4	0	1	0.00	10	1435	44	21
	Jul	2362	17912	557	1805	3755	14157	6	2	1	3.59	5	1462	57	32
	Aug	2421	17485	565	1856	3623	13862	7	2	2	3.54	2	1425	60	22
	Sep	2335	17662	569	1766	3755	13907	15	4	0	7.03	3	1492	69	28
	Oct	2336	18497	586	1750	4022	14475	7	5	1	8.53	3	1380	75	33
	Nov	2373	17658	561	1812	3897	13761	4	4	2	7.13	6	1364	41	17
	Dec	2321	17971	556	1765	3935	14036	4	1	0	1.80	9	1303	54	21

Supplemental Table 4. Clinical isolates available for sequencing and completed sequencing in the pre- and post-intervention periods.

Study period (Dates)	Clinical isolates collected/eligible	Isolates sequenced	WGS results included in analysis*
Pre-intervention (January 2019 – August 2019)	151	125	111
Post-intervention (January 2022 – December 2022)	289	289	276

* WGS, whole genome sequencing; sequenced isolates may not have passed quality control or had sufficient data for sequence analysis.

Supplemental Figure 2. Methicillin-resistant Staphylococcus aureus healthcare-associated infections in intensive care units (ICU), before and after discontinuation of contact precautions

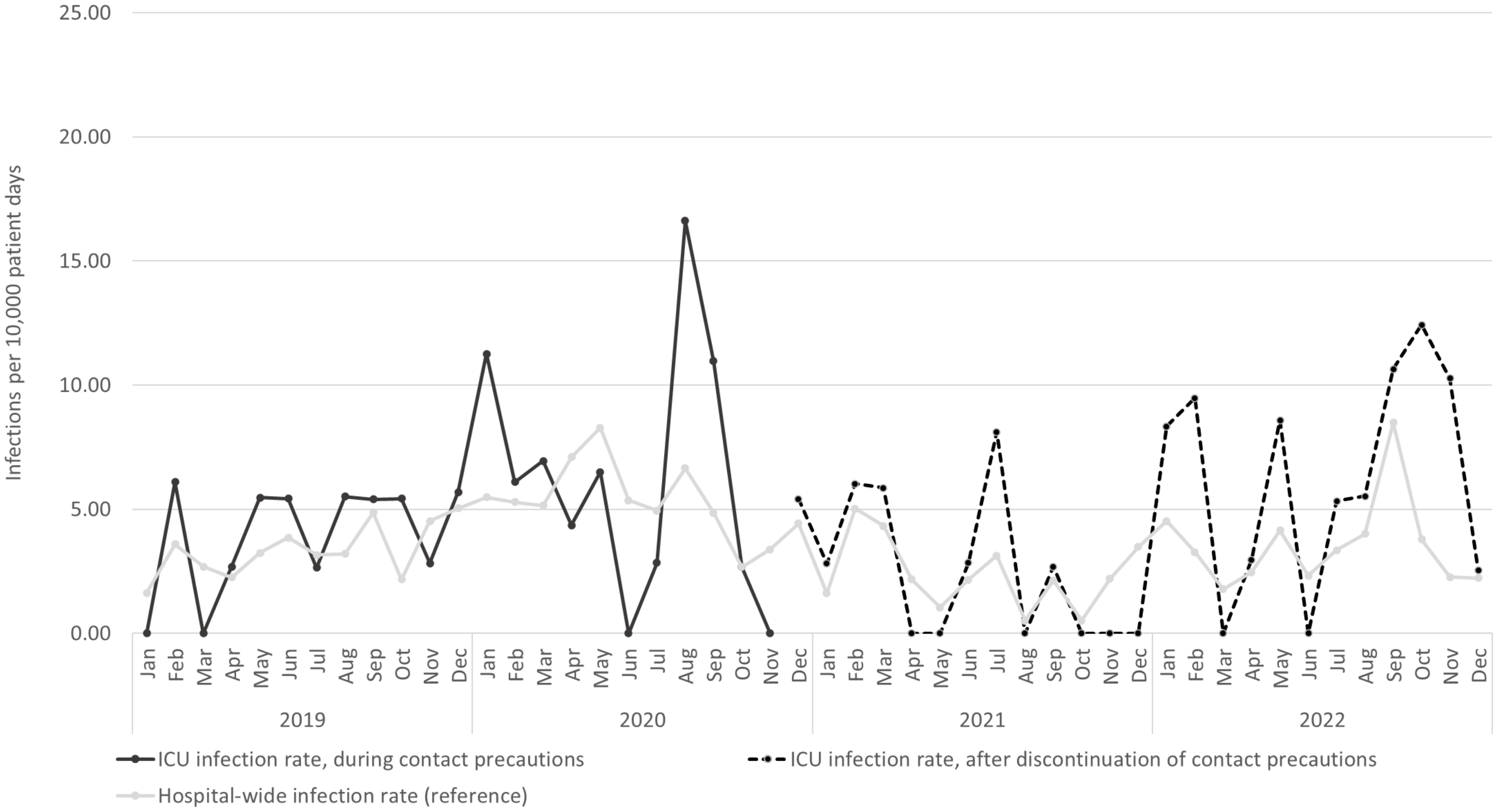

Supplemental Figure 3. Methicillin-resistant Staphylococcus aureus acquisition by active surveillance, before and after discontinuation of contact precautions

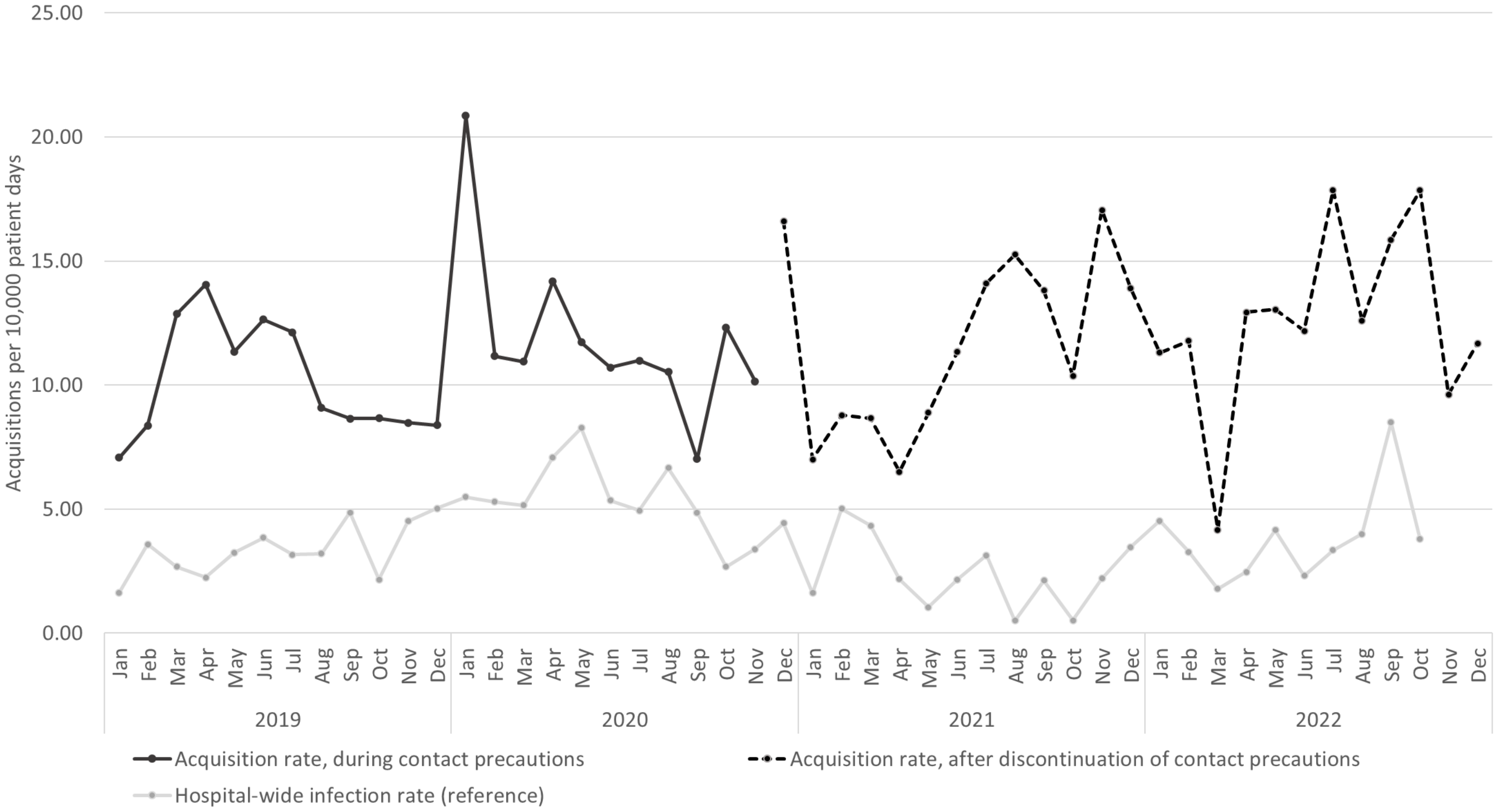

Supplemental Figure 4. Vancomycin-resistant *Enterococcus sp* healthcare-associated infections during the study period

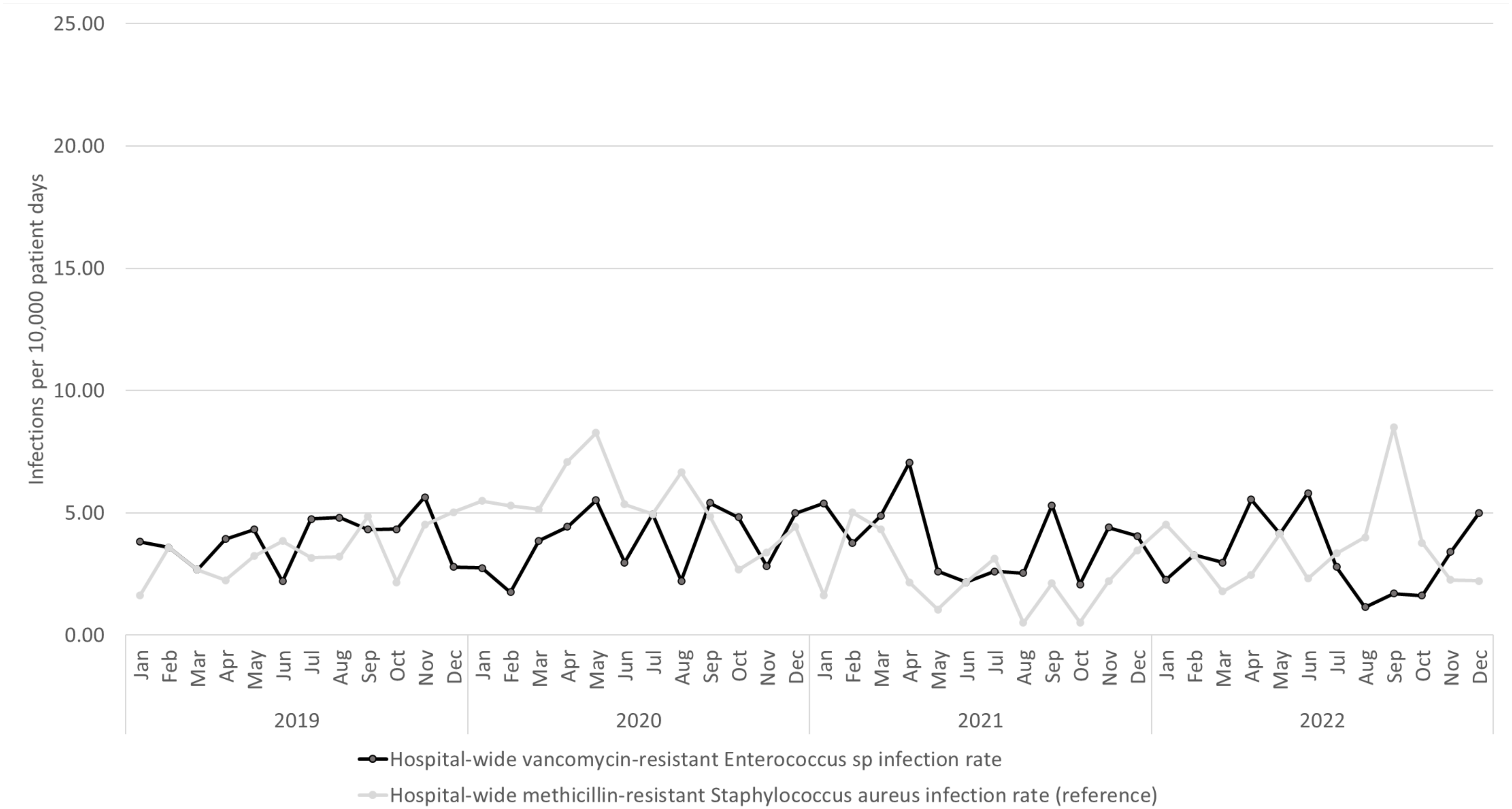

Supplement: Karunakaran et al. supplementary material [file S2732494X24000895sup001.pdf]
